# DNA Fingerprinting of Pearls to Determine Their Origins

**DOI:** 10.1371/journal.pone.0075606

**Published:** 2013-10-09

**Authors:** Joana B. Meyer, Laurent E. Cartier, Eric A. Pinto-Figueroa, Michael S. Krzemnicki, Henry A. Hänni, Bruce A. McDonald

**Affiliations:** 1 Department of Environmental System Science, Swiss Federal Institute of Technology, Zurich, Switzerland; 2 Swiss Gemmological Institute SSEF, Basel, Switzerland; 3 Department of Environmental Sciences, University of Basel, Basel, Switzerland; 4 Department of Ecology and Evolution, University of Lausanne, Lausanne, Switzerland; 5 GemExpert, Basel, Switzerland; Natural History Museum of Denmark, University of Copenhagen, Denmarkz

## Abstract

We report the first successful extraction of oyster DNA from a pearl and use it to identify the source oyster species for the three major pearl-producing oyster species *Pinctada margaritifera*, *P. maxima* and *P. radiata*. Both mitochondrial and nuclear gene fragments could be PCR-amplified and sequenced. A polymerase chain reaction-restriction fragment length polymorphism (PCR-RFLP) assay in the internal transcribed spacer (ITS) region was developed and used to identify 18 pearls of unknown origin. A micro-drilling technique was developed to obtain small amounts of DNA while maintaining the commercial value of the pearls. This DNA fingerprinting method could be used to document the source of historic pearls and will provide more transparency for traders and consumers within the pearl industry.

## Introduction

**Figure 1:**
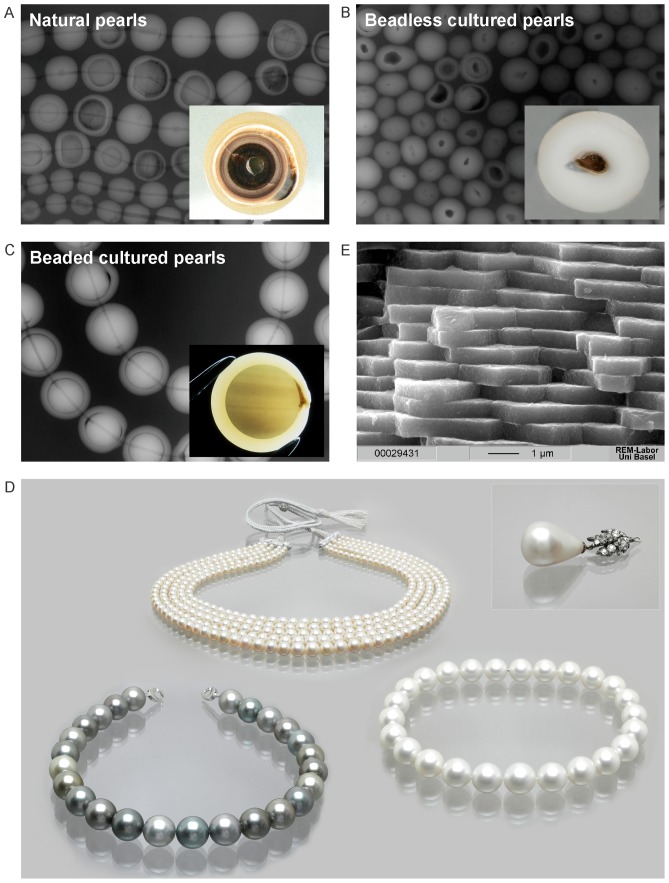
Pearls of *Pinctada margaritifera*, *P. maxima* and *P. radiata*. A) Natural pearls (*P. radiata*): radiography of a necklace and a cross-section of a pearl showing the three layers: the periostracum rich in organic material (OM) (inner layer), the prismatic layer (middle layer), and the aragonitic nacre or mother of pearl layer (outer layer). B) Beadless (without a nucleus) cultured pearls also called ‘Keshi’ (*P. maxima*): radiography of a necklace and a cross-section showing the nacreous layer around an inner cavity filled with OM. C) Beaded cultured pearls: radiography of a necklace with *P. margaritifera* pearls and cross section of an Akoya pearl showing the nacreous layer around an internal nucleus and an OM “pocket” on the right (Photos and radiographies A–C: H.A. Hänni). D) Necklaces with *P. margaritifera* pearls (lower row left), *P. radiata* pearls (upper row) and *P. maxima* pearls (lower row right). The inset shows the historic natural pearl “the Peregrina” which was found in the 16th century. This pearl and its necklace were sold for $11.8 million at a Christie's auction in December 2011 in New York. The PCR-RFLP method described here could provide scientific validation of the provenance of historic pearls (Photos: Swiss Gemmological Institute SSEF). E) Scanning electron microscope side-view image of aragonite tablets of the nacreous layer of a *P. margaritifera* pearl (Photo: Marcel Düggelin, ZMB, Basel University).

Pearls produced by oysters of the Pteriidae family are among the most valuable and oldest gems. Oyster shells and pearls have been used for human adornment since antiquity [1], [2], [3], [4], [5], [6]. Today pearls are cultured in domesticated saltwater oysters and freshwater mussels and have become a billion dollar industry [7]. Whereas a natural pearl forms without any human intervention in a wild oyster, a cultured pearl is the result of a human-induced injury. The value assigned to a pearl depends largely on its quality, rarity, and whether it originated naturally or through culture [8]. Thus there is significant interest in being able to scientifically document the provenance of both historic natural pearls [8], [9] and modern cultured pearls. This is rarely possible for the most valuable white to slightly cream-colored pearls using current methods such as UV-visible photospectrometry and micro-Raman spectroscopy [10], [11], [12], [13]. The higher value of natural pearls has led to many fraudulent attempts to pass off cultured pearls as natural ones [14], [15], [16]. To date, the distinction between natural and cultured pearls has been based on X-ray shadow images (Fig. 1A, Fig. 1B and Fig. 1C) and more recently X-ray computer microtomography [15]. Other acts of fraud involve using cultured pearls from *Pinctada maxima* and *P. margaritifera* to resemble natural pearls from *P. radiata* [17]. Although all three types of oysters have been fished for centuries in the quest for natural pearls, those from *P. radiata* from the Arabian/Persian Gulf (“Basra Pearls”) have traditionally been the most coveted [6].

Marine cultured pearls are produced mainly in three species of oysters: *P. margaritifera*, *P. maxima* and the Akoya pearl oyster (*P. fucata-imbricata*-*martensii-radiata* complex) (Fig. 1D). The *P. maxima* oysters that produce white and golden South Sea cultured pearls are found in Australia, Burma, Indonesia and the Philippines [6], [7], [18]. Pearls from *P. margaritifera* are called black cultured pearls (or Tahitian cultured pearls) and are now produced mainly in French Polynesia, Fiji, Cook Islands and Micronesia [7], [19], [20], [21]. Akoya cultured pearls are produced mainly in China, Japan and Vietnam [6], [7]. Pearls from *P. radiata* are cultured exclusively in the Arabian/Persian Gulf. The majority of natural pearls come from *P. radiata* oysters, due to a long history of pearl fisheries in the Arabian/Persian Gulf [22]. Although they play a smaller role in the natural pearl trade, *P. maxima* and *P. margaritifera* oysters have produced many natural pearls of considerable size over the last centuries [4], [23], [24]. Natural pearls have a very small niche market and remain very rare because of extremely limited production in recent decades [8].

A cultured pearl consists of nacreous aragonite (calcium carbonate, CaCO_3_) tablets (Fig. 1E) bound by an organic matrix that covers a nucleus typically made from freshwater mussel shell material (Fig. 1C and Fig. 1D) [25], [26]. A cultured pearl results from a surgical operation that subjects the oyster to a human-induced injury. After a marine pearl oyster has reached a suitable size, a small piece of external mantle tissue from a donor oyster is inserted along with a nucleus (a spherical piece of mussel shell, also called bead) (Fig. 1C) into a host oyster's gonad. The grafted mantle cells form a pearl sac that is responsible for secreting and enveloping the implanted material with aragonite, ultimately resulting in a pearl [27], [28]. The growth of a cultured pearl usually takes 6–24 months during which the cultured pearl obtains a nacreous overgrowth between 0.5 mm and 2 mm [7].

The nacreous part of a pearl consists of approximately 92% CaCO_3_, 4% organic matter (OM), 4% water and minute amounts of residual substances [29]. The OM (consisting mostly of conchioline and porphyrines), which is also secreted by the pearl sac, serves as a framework for the CaCO_3_ matrix (Fig. 1E) during the biomineralization process [30]. OM can also be found in concentrated pockets (Fig. 1C). Up until now, DNA has not been extracted from a pearl's OM, but proteins have been extracted and analyzed [31], [32], [33]. Earlier reports of DNA recovery were from calcified mussel shells [34] and the ligament that holds the valves together [35]. DNA has also been extracted from other organic gems and CaCO_3_ material (e.g. bones and teeth, corals, eggshells, ivory) [36], [37], [38], [39], [40], [41].

The aim of this research was to develop a DNA-based method to determine the oyster species that produced a pearl as a first step towards providing more precise information regarding its likely geographical origin. The DNA fingerprinting technique described here can be used to differentiate pearls from different oysters that were deliberately or accidentally mixed and may eventually differentiate cultured pearls that have been mixed in with natural pearls. DNA fingerprints could also establish the provenance of historic pearls such as the “Peregrina” pearl shown in Fig. 1D. Here we demonstrate that DNA can be extracted from a pearl's OM and used to determine the oyster species that produced the pearl. We developed a micro-drilling technique to extract the DNA that will not affect the commercial value of a pearl. These new methods will provide many advantages to the international pearl industry.

## Results and Discussion

### Pearls contain DNA that allows assignment of source *Pinctada* species

We developed a DNA extraction method from pearls to allow us to identify the *Pinctada* species that produced the pearl. We considered a DNA extraction to be successful when at least one of the four target loci was amplified by PCR and correctly identified the source *Pinctada* species. The target loci included the two mitochondrial, 16S ribosomal (rRNA) and cytochrome oxidase subunit I (*cox1*), and the two nuclear internal transcribed spacers ITS1 and ITS2. These genes were chosen because they are commonly used in oyster phylogenetic studies and are known to be variable among *Pinctada* species [42], [43], [44], [45], [46], [47], [48], [49], [50], [51], [52].

**Figure 2:**
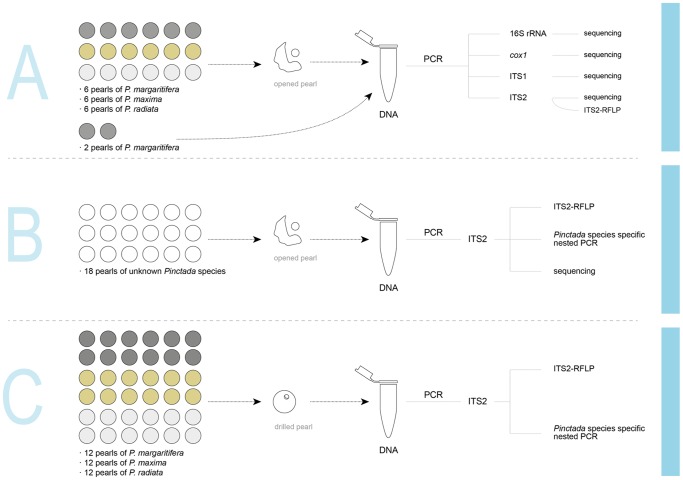
Schematic representation of the experimental procedures used for DNA extraction and PCR amplicon analysis. In methods A and B pearls were broken open using forceps to expose the internal organic material and nacre (mother of pearl). In method C samples were obtained by drilling a 1-mm diameter hole through the pearls and the hole was enlarged internally using a 0.9 mm drill head.

The *Pinctada* species were successfully identified for 100% of tested pearls from *P. margaritifera* (7/7 pearl samples) and *P. radiata* (6/6) and 60% of pearls from *P. maxima* (3/5) (Table 1 and Table 2) using method A (Fig. 2A). One pearl (PMX4) that was predicted to be *P. maxima* based on morphological criteria was instead associated to *P. margaritifera* by ITS2 and 16S rRNA sequences. The reason for this mismatch is explained below. The recovery of sequences up to 675 bp in length (Table 1) indicates that DNA is well preserved in pearls even when pearls were harvested years earlier and stored for several years at normal atmospheric conditions in a drawer or safe. The OM present in the CaCO_3_ matrix in a pearl might be a source of DNA (Fig. 1C and Fig. 1E) [53], [54]. The negatively charged DNA molecule is known to have a high affinity for the Ca^2+^ ion of CaCO_3_ [55], [56], [57], which might enhance its conservation in organic gems such as pearls. DNA recovery has been reported for several ancient CaCO_3_ materials, including eggshells from the Holocene, horse bones from the Pleistocene and other ancient bones and teeth [38], [39], [40], [58].

**Table 1:** DNA profiles of pearl samples from *Pinctada margaritifera* (PMR), *P. maxima* (PMX) and *P. radiata* (PR) based on four different molecular markers.

Pearl sample	Pearl weight (carats/mg)	Sample weight (mg)	16S rRNA	*cox1*	ITS1 rRNA	ITS2 rRNA
PMR positive control			PMR (AB214436.1)^a^	PMR (AB259166.1)	PMR (AY877501.1)	PMR (AY877506.1)
			511 bp (99%)^b^	575 bp (99%)	675 bp (99%)	575 bp (100%)
PMX positive control			PMX (AB214435.1)	PMX (GQ452847.1)	PMX (AY172345.1)	PMX (AY877505.1)
			509 bp (100%)	476 bp (99%)	593 bp (99%)	571 bp (100%)
PR positive control			PR (AB214442.1)	PR (GQ355875.1)	*P. martensii* c (AY172344.1)	*P. fucata* c (AY877582.1)
			524 bp (100%)	575 bp (99%)	580 bp (99%)	591 bp (99%)
PMR1	11.1/2228	426	PMR (AB214436.1)	PMR (AF374329.1)	PMR (AY877501.1)	PMR (AY877506.1)
			511 bp (99%)	425 bp (99%)	675 bp (99%)	575 bp (100%)
PMR2	8.1/1610	19	PMR (AB214436.1)	PMR (AF374329.1)	PMR (AY877501.1)	PMR (AY877506.1)
			455 bp (99%)	425 bp (99%)	378 bp (100%)	575 bp (100%)
PMR3	7.4/1480	24	n.d.^d^	n.d.	n.d.	PMR (AY877506.1)
						575 bp (100%)
PMR4	7.4/1480	124	PMR (AB214436.1)	PMR (AF374329.1)	PMR (AY877501.1)	PMR (AY877506.1)
			455 bp (99%)	425 bp (99%)	378 bp (100%)	575 bp (100%)
PMR5	13.1/2618	318	PMR (AB214436.1)	PMR (AF374329.1)	n.d.	PMR (AY877506.1)
			455 bp (100%)	425 bp (99%)		575 bp (100%)
PMR6	9.8/1964	23	PMR (AB214436.1)	PMR (AF374326.1)	n.d.	PMR (AY877506.1)
			454 bp (99%)	425 bp (100%)		575 bp (100%)
PMX1	33.0/6598	78	PMX (AB214435.1)	n.d.	n.d.	PMX^e^
			451 bp (100%)			
PMX2	29.5/5898	135	PMX (AB214435.1)	n.d.	n.d.	PMX (AY883851.1)
			451 bp (100%)			571 bp (100%)
PMX3	20.9/4180	34	PMX (AB214435.1)	PMX (GQ452847.1)	n.d.	PMX (AY282737.1)
			451 bp (100%)	204 bp (100%)		571 bp (100%)
PMX4	25.3/5070	105	PMR (AB214436.1)	n.d	n.d.	PMR (AY877506.1)
			454 bp (99%)			575 bp (99%)
PMX5	13.5/2694	38	n.d.	n.d.	n.d.	n.d.
PMX6	8.4/1672	59	n.d.	n.d.	n.d.	n.d.
PR1	6.2/1234	108	PR (AB214442.1)	n.d.	*P. martensii* (AY144602.1)	*P. fucata* (AY877582.1)
			444 bp (100%)		226 bp (99%)	590 bp (99%)
PR2	5.4/1090	79	PR (AB214442.1)	PR (GQ355875.1)	*P. martensii* (AY144602.1)	*P. fucata* (AY877588.1/AY877600.1)^f^
			444 bp (100%)	543 bp (99%)	226 bp (99%)	221 bp/239 bp (100%)
PR3	5.1/1030	296	PR (AB214442.1)	n.d.	n.d.	*P. fucata* (AY877582.1)
			523 bp (100%)			491 bp (99%)
PR4	4.5/908	224	PR (AB214442.1)	PR (GQ355875.1)	*P. martensii* e	*P. fucata* (AY877588.1/AY877600.1)^f^
			523 bp (100%)	543 bp (99%)		221 bp/239 bp (100%)
PR5	4.5/904	151	n.d.	*P. fucata* (DQ299941.1)	n.d.	*P. fucata* (AY877582.1)
				149 bp (91%)		491 bp (99%)
PR6	4.2/842	83	PR (AB214442.1)	PR (GQ355875.1)	n.d.	*P. fucata* (AY877605.1)
			523 bp (100%)	543 bp (99%)		242 bp (99%)

a *Pinctada* species assignment was based on the highest BLAST score (highest query coverage and maximal base pair identity). GenBank accession number shown in brackets.

b amplicon size (base pair) and maximal identity (%) of the sequence to the BLAST query.

c *P. fucata* and *P. martensii* are conspecific to *P. radiata* on the basis of their ITS sequences [50], [51].

d not determined.

e sample had lower sequence quality, but the BLAST query in GenBank indicated the correct *Pinctada* species. The ITS2 sequences could be amplified and successfully analyzed using PCR-RFLP.

f these two accession numbers correspond to ITS2 sequences which flanked an internal sequence of ∼30 bp characterized by double peaks consistent with heterozygosity.

**Table 2:** Sequencing success rate associated with different molecular markers from pearl DNA extracts of *Pinctada margaritifera*, *P. maxima* and *P. radiata* using methods A, B and C (Fig. 2).

Method A^a^	16S rRNA	*cox1*	ITS1	ITS2	Total % of successfully identified pearls
*P. margaritifera*	86% (6/7)^b^^, ^ c	71% (5/7)	43% (3/7)	100% (7/7)	100% (7/7)^c^
*P. maxima*	60% (3/5)^c^	20% (1/5)	0% (0/5)	60% (3/5)	60% (3/5)^c^
*P. radiata*	83% (5/6)	67% (4/6)	50% (3/6)	100% (6/6)	100% (6/6)
Total % of successfully sequenced markers	78% (14/18)	56% (10/18)	33% (6/18)	89% (16/18)	89% (16/18)

Methods A, B and C^a^	Method A^a^	Method B^a^	Methods A+B^a^	Method C^a^ practically “non-destructive”
	ITS2	ITS2	ITS2	ITS2
*P. margaritifera*	100% (7/7)^b^^, ^ c	100% (7/7)^c^	100% (14/14)^c^	92% (11/12)
*P. maxima*	60% (3/5)^c^	80% (4/5)^c^	70% (7/10)^c^	58% (7/12)
*P. radiata*	100% (6/6)	100% (6/6)	100% (12/12)	92% (11/12)
Total % of successfully sequenced markers	89% (16/18)	94% (17/18)	92% (33/36)	81% (29/36)

a in methods A and B the pearls were broken open using forceps to expose the inner material used to extract DNA. In method C the powder used for DNA extraction was obtained by drilling a 1-mm diameter hole in the pearls and the hole was enlarged internally using a 0.9 mm drill head.

b percentage (%) of successfully identified pearls (identified pearls/total pearls tested).

c from a total of twelve *P. maxima* and *P. margaritifera* samples analyzed in method A or in method B, one pearl that was predicted to belong to *P. maxima* based morphological criteria was identified as *P. margaritifera* according to the DNA fingerprint.

Mitochondrial genes are present at a higher copy number per cell than nuclear genes and are thought to degrade more slowly due to their organellar location [59]. Thus they are often preferentially targeted in degraded, ancient and diluted samples [58], [59]. Nevertheless, we had greater success amplifying and sequencing the nuclear ITS2 gene than the mitochondrial 16S rRNA or *cox1* genes. These results suggest that the DNA is well preserved in the interior of the pearl.

Complete ITS2 sequences were obtained for *P. margaritifera* and *P. maxima* (Table 1), but two of the *P. radiata* samples (PR2 and PR4) had ∼30 bp of internal sequence characterized by double peaks consistent with heterozygosity in this small region (Table 1). Intra-individual ITS polymorphism is common in oyster species [47], [49], [51]. Moreover, because cultured pearls are formed by grafting nacre-secreting mantle tissue from a donor oyster into the gonad of a recipient oyster (host), the two organisms might have different ITS sequences that will be mixed in the pearl [60]. Sequence polymorphisms were found among *P. margaritifera* pearls in mitochondrial 16S rRNA and *cox1* sequences as well as in the ITS2 sequence of PMX4. No polymorphisms were detected among *P. maxima* pearls. DNA sequences were deposited in GenBank under accession numbers KF283999–KF284026 (ITS1 and ITS2), KF284042–KF284058 (16S rRNA) and KF284059–KF284070 (*cox1*).

None of the four loci could be amplified from the *P. maxima* pearls PMX5 and PMX6 (Table 1). Pearl PMX5 contained a malodorous brown liquid consistent with degradation of the OM and possibly degradation of the corresponding DNA. Other *P. maxima* pearls generally contained little visible OM and had thinner and more resistant outer nacreous layers around the internal nucleus. *P. margaritifera* and *P. radiata* pearls were characterized by a relatively higher visible OM content, which was correlated with higher PCR amplification success. We had successful amplification from samples composed only of white powder, indicating that DNA can be obtained through demineralization from the CaCO_3_ structure (Fig. 1) of the nacre and/or from small samples (e.g.: PMR2 = 19 mg, Table 1).

We failed to amplify any DNA from the two intact pearls of *P. margaritifera* (pearls PMRA and PMRB, Fig. 2A) that were not broken open before adding them to the ethylenediaminetetraacetic acid (EDTA) buffer. Pearls are often washed with freshwater and cleaned using salt or ground up walnut shells to remove surface impurities, and some pearls can be treated using, for example, the maeshori method that involves the use of solvents such as methyl alcohol [61]. Moreover, we sterilized the pearls for 20 min in a sodium hypochlorite solution prior to DNA extraction. These treatments may explain why we could not extract DNA from the outer layer. The minimal surface area exposed to the EDTA might also have hampered DNA extraction. Other studies showed that recovery of DNA from freshwater shell material of *Margaritifera margaritifera* was strongly affected by exposure time and grinding intensity [34]. We did not further develop testing procedures for entire pearls because this totally destructive method would not be acceptable in the pearl trade. We therefore focused our efforts on developing the less destructive micro-drilling method described later in this paper.

### A PCR-RFLP test to determine pearl origins

**Figure 3:**
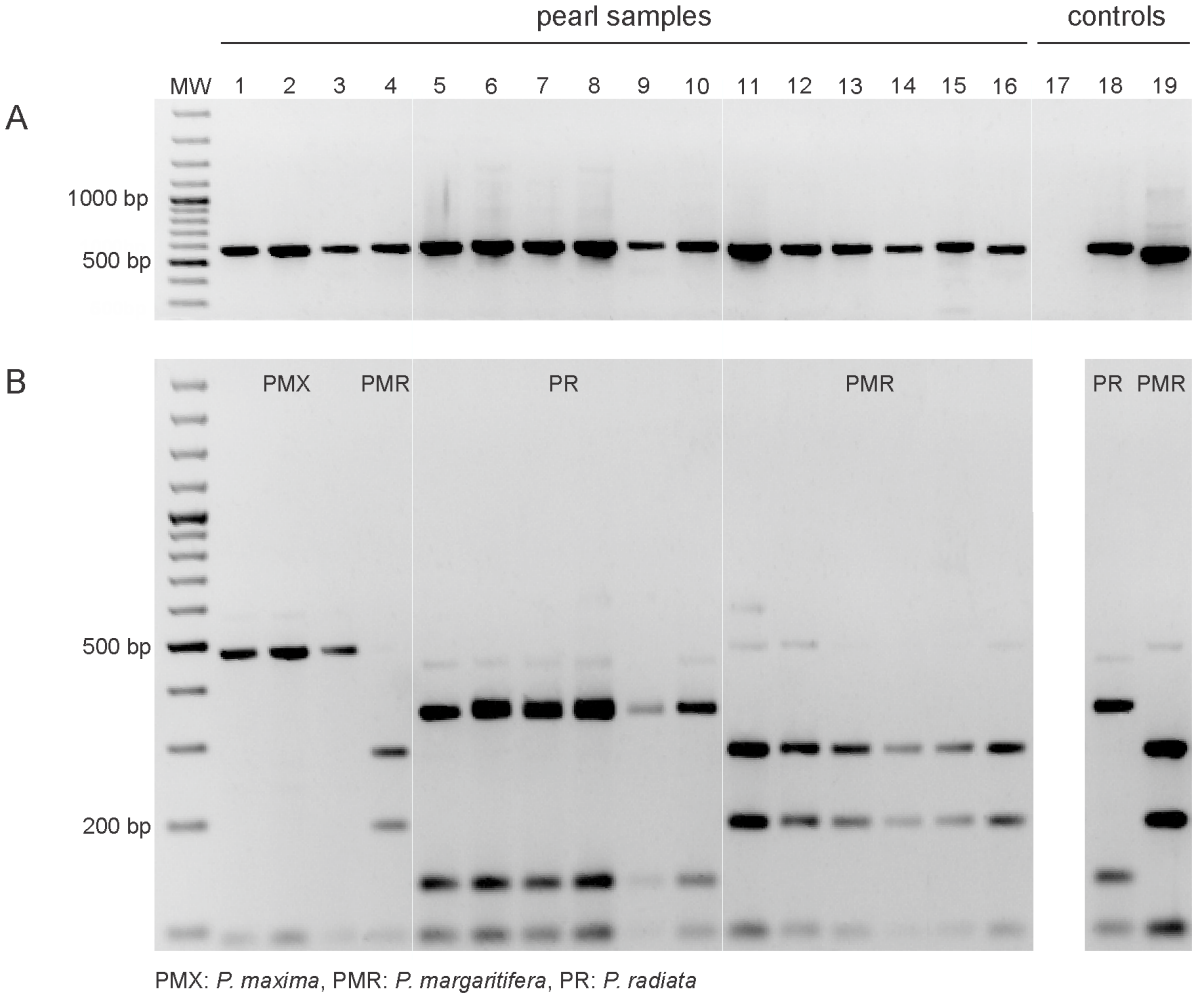
A PCR-RFLP assay of the ITS2 region applied to pearls from *Pinctada margaritifera*, *P. maxima* and *P. radiata*. (A) PCR products of 575 bp (*P. margaritifera*), 571 bp (*P. maxima*) and 590–91 bp (*P. radiata*) obtained with ITS2 universal primers (5.8S-F and 28S-R) and (B) RFLP patterns of ITS2 amplicons (from A) obtained after digestion with *Rsa*I. MW: molecular weight size marker, 100-bp DNA ladder; lanes 1–3: *P. maxima* (PMX) pearls; lane 4: *P. margaritifera* (PMR) pearl; lanes 5–10: *P. radiata* (PR) pearls; lanes 11–16: *P. margaritifera* pearls; lane 17: PCR negative control; lanes 18 and 19: *P. radiata* and *P. margaritifera* positive controls. Note: The *P. maxima* positive control is shown in Figure 4.

Sequences of ITS regions have been widely used to differentiate *Pinctada* species [47], [49], [51], [52] and an RFLP method has already been developed on the intergenic spacer (IGS) of nuclear ribosomal RNA to distinguish the closely related *P. fucata*, *P. imbricata* and *P. martensii* [49]. We developed a PCR-RFLP method based on the ITS2 region to differentiate among the three examined *Pinctada* species (Fig. 3).

**Figure 4:**
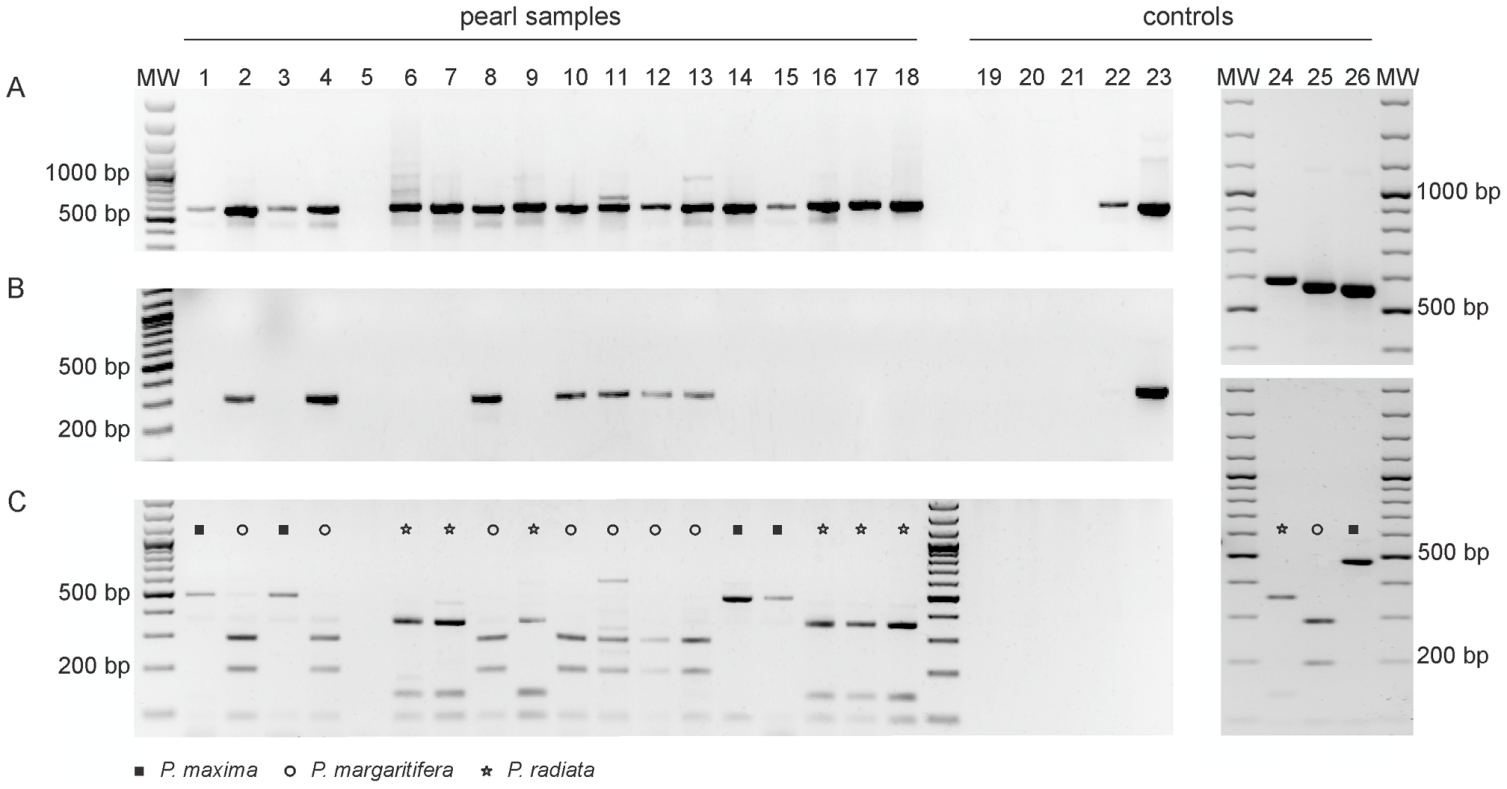
Blind PCR-RFLP assay with eighteen pearls of unknown identity. (A) PCR products of 575 bp (*Pinctada margaritifera*), 571 bp (*P. maxima*) and 590–91 bp (*P. radiata*) obtained with ITS2 universal primers (5.8S-F and 28S-R) and (B) of 335 bp obtained with 28S-R and the *P. margaritifera* specific primer ITS2-Marg-F. (C) RFLP patterns of ITS2 gene fragments (from A) obtained after digestion with *Rsa*I. MW: molecular weight size marker, 100 bp DNA ladder; lanes 1–18: pearl isolates; lanes 19–20: DNA extraction negative controls; lane 21: PCR negative control; lanes 22–23: *P. radiata* and *P. margaritifera* positive controls; lanes 24–26: *P. radiata*, *P. margaritifera* and *P. maxima* positive controls showing ITS2 PCR products (upper gel) and ITS2-RFLP patterns (lower gel).

To validate the PCR ITS2-RFLP method, 18 pearls of unknown identity were included in a blinded analysis (Fig. 2B). ITS2 was successfully amplified from 17 out of 18 pearls (Fig. 4A, Table 2). PCR with *P. margaritifera* specific primers amplified only the corresponding *P. margarifiera* pearl samples (Fig. 4B) and the PCR ITS2-RFLP analysis allowed us to correctly identify each pearl (Fig. 4C) except for BL4 that we identified as *P. margaritifera* instead of *P. maxima*. As explained below, we consider the PCR ITS2-RFLP assay to be more accurate than the conventional assay based on morphological criteria. The results of the PCR ITS2-RFLP assay were confirmed by sequencing the ITS2 region amplified in each pearl (GenBank accession numbers KF284027–KF284041; Table S1). The method was successful across a variety of pearls of different sizes, shapes and composition of the extracted material (weight range from 38 mg to 672 mg) (Table S1).

### Potential applications in the pearl industry

**Figure 5:**
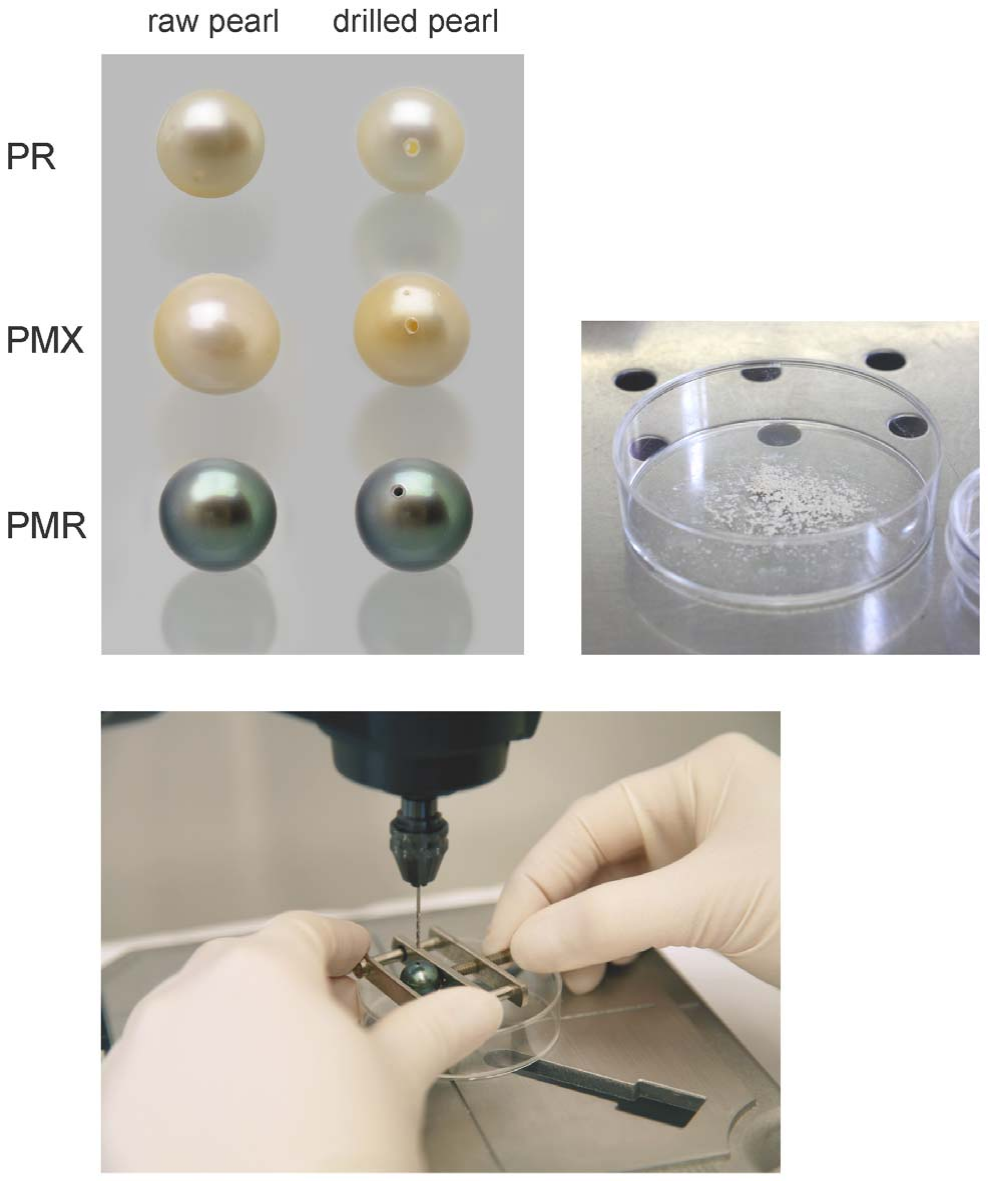
Examples of pearls of *Pinctada margaritifera*, *P. maxima* and *P. radiata* used in this study before and after micro-drilling. We used a drill head attached to a Dremel Workstation to produce pearl powder used for DNA extraction. Recovered pearl powder (nacre and organic material) can be seen in the Petri dish. *P. margaritifera* (PMR), *P. maxima* (PMX) and *P. radiata* (PR).

To minimize the potential loss in pearl value that would result from damaging the pearl to obtain sufficient material for a DNA test, we developed a micro-drilling methodology (Fig. 5) that could be especially useful for determining the origin of historic natural pearls of high value (see for example Fig. 1D). We tested this method on twelve pearls for each *Pinctada* species (Fig. S1 and Fig. 2C). For both *P. margaritifera* and *P. radiata*, 11 out of 12 pearls could be successfully identified using as little as 10 mg of recovered drill powder (Table 2 and Table 3). For *P. margaritifera* it was possible to amplify the ITS2 with a direct PCR, but in *P. radiata* and *P. maxima* a nested PCR approach using an additional specific primer internal to the ITS2 region was needed. All of our experiments indicate that DNA recovery is more difficult from *P. maxima* than the other species.

**Table 3:** ITS2 profiles of pearls from *Pinctada margaritifera* (PMR), *P. maxima* (PMX) and *P. radiata* (PR) using a practically non-destructive method (Fig. 2C).

Pearl label	Pearl weight (carats/mg)	Sample weight (mg)	ITS2 direct PCR^a^	ITS2 nested PCR^a^	ITS2-RFLP	PMR, PMX or PR ITS2 nested PCR^a^
PMR7	6.7/1335	43	no	no	no	no
PMR8	7.5/1511	45	***yes***	***yes***	***yes***	***yes***
PMR9	7.9/1588	60	***yes***	***yes***	***yes***	***yes***
PMR10	12.2/2441	61	***yes***	***yes***	***yes***	***yes***
PMR11	11.5/2307	59	***yes***	***yes***	***yes***	***yes***
PMR12	9.7/1934	59	***yes***	***yes***	***yes***	***yes***
PMR13	10.2/2048	74	***yes***	***yes***	***yes***	***yes***
PMR14	6.5/1310	75	***yes***	***yes***	***yes***	***yes***
PMR15	15.9/3190	50	***yes***	***yes***	***yes***	***yes***
PMR16	12.3/2464	39	***yes***	***yes***	***yes***	***yes***
PMR17	6.7/1335	71	***yes***	***yes***	***yes***	***yes***
PMR18	7.5/1511	100	***yes***	***yes***	***yes***	***yes***
			92% (11/12)^b^	92% (11/12)	92% (11/12)	92% (11/12)
PMX7	11.6/2320	90	no	no	no	no
PMX8	15.6/3120	50	no	no	no	***yes***
PMX9	6.4/1290	20	no	no	no	***yes***
PMX10	7.2/1450	60	no	***yes***	***yes***	***yes***
PMX11	18.6/3720	110	no	***yes***	***yes***	***yes***
PMX12	20.2/4030	90	no	no	no	no
PMX13	12.4/2470	100	no	no	no	no
PMX14	17.4/3480	70	no	no	no	no
PMX15	12.0/2400	60	no	no	no	no
PMX16	12.1/2420	100	no	***yes***	***yes***	***yes***
PMX17	10.4/2080	70	no	***yes***	***yes***	***yes***
PMX18	9.3/1860	40	no	***yes***	***yes***	***yes***
			0% (0/12)^b^	42% (5/12)	42% (5/12)	58% (7/12)
PR7	6.9/1380	40	no	***yes***	***yes***	***yes***
PR8	4.9/970	20	no	***yes***	***yes***	***yes***
PR9	4.7/940	10	no	***yes***	***yes***	***yes***
PR10	6.0/1210	13	no	***yes***	***yes***	***yes***
PR11	6.1/1220	40	no	no	no	***yes***
PR12	5.4/1080	33	no	***yes***	***yes***	***yes***
PR13	6.5/1310	40	no	***yes***	***yes***	***yes***
PR14	6.2/1240	20	no	no	no	***yes***
PR15	7.0/1400	20	no	no	no	***yes***
PR16	5.2/1050	20	no	***yes***	***yes***	***yes***
PR17	4.2/850	20	no	***yes***	***yes***	***yes***
PR18	5.1/1020	20	no	no	no	no
			0% (0/12)^b^	67% (8/12)	67% (8/12)	92% (11/12)

a direct PCR was conducted using ITS2 universal primers (5.8S-F and 28S-R). Nested PCR was conducted with the universal ITS2 primers or primer pair 28S-R and *Pinctada*-specific forward primers internal to the ITS2 fragment (ITS2-Marg-F, ITS2-Max-F or ITS2-Rad-F).

b percentage of successfully identified pearls (identified pearls/total pearls tested).

### *P. margaritifera* or *P. maxima*, which method is more accurate?

An unexpected outcome was the mixed identity assigned to the cultured pearls PMX4 and BL4 (Table 1 and Table S1, Fig. 3 and Fig. 4). These pearls were assigned to the *P. maxima* species by pearl experts at the Swiss Gemmological Institute SSEF through visual observation, mainly because of their cream color. However, their DNA fingerprints (PCR ITS2-RFLP and sequences of 16S rRNA and ITS2) clearly indicated that these pearls originated from *P. margaritifera*. The ITS2 sequence of PMX4 differed from *P. margaritifera* by only two single nucleotide polymorphisms (Table 1). Based on our overall results, we believe that the visual assignment of species origin was incorrect, as it is well known that *P. margaritifera* not only produces grey to black pearls, but also yellowish to white ones, which are very similar in color to pearls from *P. maxima* [10], [19]. A recent study [45] found a Japanese *P. maxima* oyster, identified based on its morphology clustering with *P. margaritifera*, on the basis of its *cox1* sequence and concluded that the mismatch was due to inaccuracy of the morphological measurement. Similarly, a specimen identified as *P. radiata* on the basis of morphology had an ITS1 sequence matching *P. chemnitzi* [51]. These mistaken identifications based on morphology illustrate well the need for an accurate method to determine the origins of pearls produced by *Pinctada* oysters.

## Conclusions

We were able to extract DNA from individual pearls and develop a PCR-RFLP assay to determine which oyster species produced the pearl. This method can potentially be used to document the provenance of historic pearls and determine which oyster species produced either natural or cultured pearls. The ability to extract relatively large DNA molecules from pearls opens the possibility of applying next generation DNA sequencing (NGS) technologies [38] to provide more extensive sequence data that would provide even more precise information on pearl origins. We anticipate that NGS technologies coupled with detailed population genetic analyses of reference oyster populations could enable individual pearls to be assigned to specific oyster populations, allowing a scientific assignment of a pearl's origin and providing more transparency for traders and consumers within the pearl industry.

## Materials and Methods

### Animal sample preparation and DNA extraction

Three oyster specimens each of *P. margaritifera*, *P. maxima* and *P. radiata* were collected at pearl farms in Pohnpei (Federated States of Micronesia) in December 2011, Bali (Indonesia) in May 2013 and Ras Al Khaimah (United Arab Emirates) in January 2012 and stored at −20°C. A 0.5–1.0 g piece of adductor muscle was ground in liquid nitrogen and total genomic DNA was extracted according to the manufacturer's recommendations using the QIAGEN DNeasy® Plant Mini Kit (QIAGEN, Hilden, Germany). DNA was diluted to 10 ng/µl and stored at −20°C until further use. These DNA samples were used as positive controls for the PCR-RFLP and sequencing analyses.

### Pearl material

All samples were non-drilled marine cultured pearls of known origin. All pearls contained a nucleus (a spherical bead of freshwater mussel shell) typically used in pearl production. Natural pearls were not used because they are much more valuable and their geographic and species provenance is rarely well documented. In total, 74 pearls were studied using three different methodologies (A, B and C: see Fig. 2). For method A six pearls of each *Pinctada* species were analyzed using destructive DNA extraction methods (PMR1–6 for *P. margaritifera*, PMX1–6 for *P. maxima* and PR1–6 for *P. radiata*) and two additional *P. margaritifera* pearls, PMRA and PMRB, were analyzed non-destructively. For method B a blind test based on destructive DNA extraction was carried out using 18 pearls from an unknown source (BL1–18) that was later revealed. For method C, the DNA of 12 pearls of each *Pinctada* species (PR7–18, PMX7–18 and PMR7–18) were analyzed using micro-drill sampling (pearls are shown in Fig. S1). *P. margaritifera* pearls were collected in French Polynesia between 2007 and 2010, except nine pearls harvested in Fiji in 2010–2011 (PMRB in method A, and PMR9 to 16 in method C). *P. maxima* pearls were grown either in Australia or Indonesia and harvested between 2005–2009, except for two pearls from the Philippines, PMX16 and PMX17 (method C) harvested in 2003 and 2010, respectively. *P. radiata* pearls were harvested at pearl farms in Ras Al Khaimah (United Arab Emirates) in 2009 and 2010. Pearls were provided by RAK Pearls (United Arab Emirates) and Dr. Masahiro Ito (Pohnpei, Micronesia), Andy Müller (Kobe, Japan), Frieden AG (Thun, Switzerland) and Jörg Gellner (Zürich, Switzerland). Pearl weights ranged from 1154–3190 mg (5.8–15.9 carats) for *P. margaritifera*, from 856–6598 mg (4.3–32.9 carats) for *P. maxima* and from 504–1754 mg (2.5–8.8 carats) for *P. radiata*.

### Preparing pearls for DNA extraction

The three different DNA extraction and analysis methodologies (A, B and C) are illustrated in Fig. 2. To minimize the possibility of DNA cross contamination, DNA extraction from pearls was performed in a different laboratory room and sterile hood than DNA extraction from the adductor muscle. All pearls were surface sterilized by stirring in a 4% sodium hypochlorite solution for 20 min. For methods A and B (Fig. 2), the pearls were broken open using sterile forceps in a sterile hood, except PMRA and PMRB which were tested in their original state (i.e. as intact pearls). The inner nucleus was discarded and the remaining material was pulverized in a mortar, added to a 2 ml microfuge tube and weighed. The two intact pearls were added to 2 ml microfuge tubes and weighed. 500 µl of 0.5 M EDTA at pH 8.0, was added to each sample to dissolve the CaCO_3_. For method C (Fig. 2) the material used for DNA extraction was removed by drilling a hole using a Dremel® (Model 8000, Dremel Europe, Breda, Netherlands) with a 1 mm drill head fixed on a Dremel® Workstation (Fig. 5). The pearl was held in a vise over a sterile Petri dish that collected the resulting drill powder. A second non-fixed 0.9 mm drill head was used to enlarge the interior part of the drill hole without damaging the surface around the drill hole. The drill powder was suspended in 1000 to 2000 µl 0.5 M EDTA (pH 8.0). All pearl samples in the EDTA solution were vigorously vortexed for two min and incubated overnight at 56°C in a water bath.

### DNA extraction

Total DNA was extracted directly from the pearl-EDTA solution using a Fast DNA Spin Kit for soil (MP Biomedicals, Irvine, CA, USA). The extraction procedure was done according to the manufacturer's recommendations except that in the first step 1000 or 700 µl of sodium phosphate buffer included in the kit was directly added to the microfuge tube when it contained 500 µl or 1000 µl EDTA, respectively. When samples were incubated in 2000 µl EDTA, the sample was divided evenly into two 2 ml microfuge tubes and each tube received 700 µl of sodium phosphate buffer. The Lysing Matrix E tubes provided in the kit were not used. Homogenization with the FastPrep instrument was not performed; instead the samples were vortexed vigorously for two minutes. The resulting DNA samples were used directly, diluted ten times, or concentrated in a vacuum centrifuge prior to PCR.

### PCR amplification

DNA samples were screened for the presence of the mitochondrial-encoded 16S rRNA and the *cox1* genes and the nuclear-encoded ITS1 and ITS2 of the rRNA gene cluster. *Pinctada* ITS2 gene sequences were retrieved from GenBank and aligned using the multiple sequence alignment program ClustalW 1.8 [62]. Sequences that were polymorphic between *P. margaritifera*, *P. maxima* and *P. radiata* were used to design species-specific forward primers ITS2-Marg-F, ITS2-Max-F and ITS2-Rad-F. All primers, annealing temperatures and PCR conditions used in this study and the expected lengths of the PCR amplicons are listed in Table S2.

PCR was carried out in 20 µl reactions containing 1 µl of DNA template, 2 µl of PCR buffer (Fermentas GmbH, St. Leon-Rot, Germany), 5% bovine serum albumin (New England Biolabs, Inc., Beverly, MA), 5% dimethylsulfoxide (Sigma-Aldrich Chemie GmbH, Buchs, Switzerland), 200 µM of each dATP, dCTP, dGTP, and dTTP (New England Biolabs, Inc.), 0.50 µM of each primer and 1.4 U of Dream DNA polymerase (Fermentas GmbH). The initial denaturation (5 min at 94°C) was followed by 40 cycles of 94°C for 30 s, as annealing temperature of 45–55°C for 30 s and 72°C for 60 s with a final extension at 72°C for 7 mins.

### Sequencing of 16S rRNA, *cox1*, ITS1 and ITS2

All PCR amplicons were purified on a MultiScreen PCR plate (Millipore, Molsheim, France) and resuspended in 30 µl of sterile double-distilled water. Sequencing reactions were performed with 3–10 ng of purified PCR product and primers at a final concentration of 0.10 µM using an ABI PRISM BigDye Terminator v3.0 cycle sequencing kit (Applied Biosystems, Foster City, CA, USA) according to the manufacturer's instructions. PCR products were sequenced in both directions using the same primer pairs as in the amplification reaction (Table S2). The obtained products were cleaned by gel-filtration through Sephadex G-50 columns (Amersham Biosciences, Uppsala, Sweden) on MultiScreen HV plates (Millipore). Purified products were sequenced using an ABI Prism 3130 Genetic Analyzer (Applied Biosystems) at the Genetic Diversity Centre of the ETH Zürich. DNA sequences were edited using the Sequencher package (Gene Codes, Ann Arbor, MI, USA). Only the unambiguous parts of the sequence were used to define the species through homology with the NCBI Databank.

### PCR-RFLP analyses

To discriminate between *Pinctada* species, a PCR-RFLP analysis was performed on the PCR-amplified ITS2 gene fragment. Candidate restriction endonucleases were identified using the software Nebcutter 2.0 [63]. Restriction analysis was done in 12 µl reaction mixtures with 5 µl of amplified product, 100 µg/ml bovine serum albumin (New England Biolabs, Inc.), 1.2 µl enzyme buffer (New England Biolabs, Inc.) and 0.5 units of *Rsa*I (Fermentas GmbH). Reactions were incubated for 90 min at 37°C and then stored at −20°C. Restriction fragments were separated by electrophoresis in ethidium bromide-stained 2% agarose gels. A 100 bp ladder (GIBCO-BRL Life Technologies Inc., Gaithersburg, MD, USA) was used as a size marker. The digested PCR products were compared with equivalent RFLP profiles obtained from the reference positive control *P. margaritifera*, *P. maxima* and *P. radiata* adductor muscle DNA extracts.

## Supporting Information

Figure S1 Pearls from *Pinctada margaritifera* (PMR), *P. maxima* (PMX) and *P. radiata* (PR) used in method C (Fig. 2).
(PDF)

Table S1 Blind test: PCR-RFLP and analysis of the ITS2 sequences from eighteen pearls of unknown identity.
(PDF)

Table S2 PCR primers, amplicon lengths and references.
(PDF)
