# Supplementary figures and images for: DNA Fingerprinting of Pearls to Determine Their Origins
Source: PLoS One. 2013 Oct 9;8(10):e75606. doi: 10.1371/journal.pone.0075606 (PMC3794040; doi:10.1371/journal.pone.0075606)

**Figure S1.** Pearls from *P. margaritifera* (PMR), *P. maxima* (PMX) and *P. radiata* (PR) used in method C (Fig. 2).

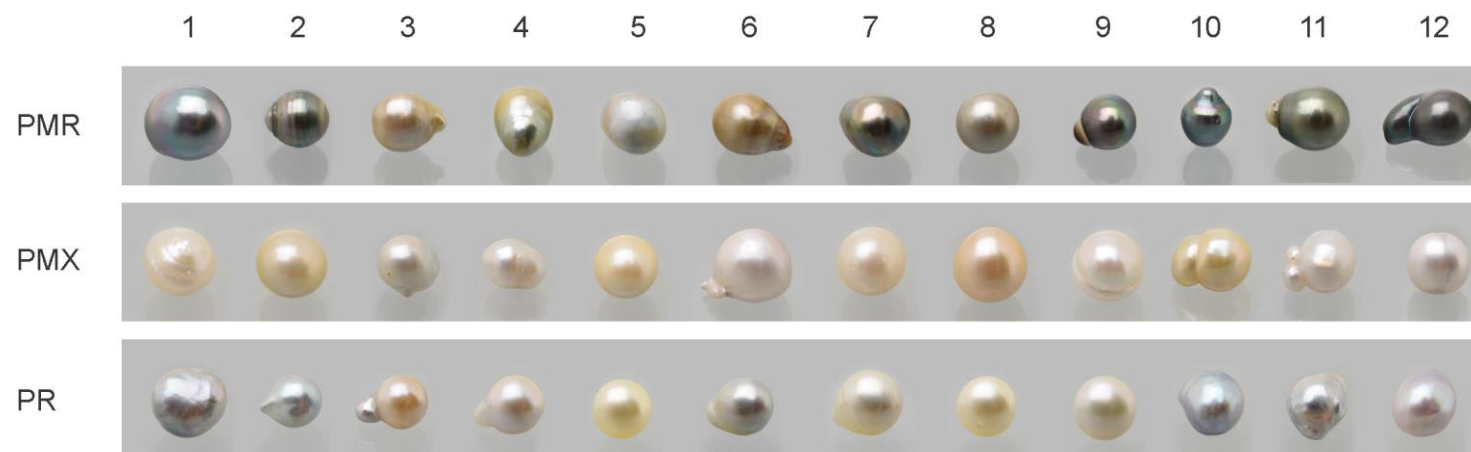

Supplement: Figure S1 — Pearls from Pinctada margaritifera (PMR), P. maxima (PMX) and P. radiata (PR) used in method C (Fig. 2). (PDF) [file pone.0075606.s001.pdf]
