# Supplementary material for: DNA Fingerprinting of Pearls to Determine Their Origins
Source: PLoS One. 2013 Oct 9;8(10):e75606. doi: 10.1371/journal.pone.0075606 (PMC3794040; doi:10.1371/journal.pone.0075606)
Supplement: Table S1 — Blind test: PCR-RFLP and analysis of the ITS2 sequences from eighteen pearls of unknown identity. (PDF) [file pone.0075606.s002.pdf]

Table S1. Blind test: PCR-RFLP and analysis of the ITS2 sequences from eighteen pearls of unknown identity.

Pearl sample	Pearl weight (carats / mg)	Sample weight (mg) [a]	ITS2-RFLP [b] results	ITS2 [b] rRNA sequence match	GenBank accession number	Correctly identified?
BL1	16.8 / 3358	103	*P. maxima*	*P. maxima* (AY883851.1) [c] 461 bp (100 %) [d]	KF284027	*yes*
BL2	10.5 / 2092	158	*P. margaritifera*	*P. margaritifera* (AY877506.1) 292 bp (100 %)	KF284028	*yes*
BL3	10.1 / 2012	61	*P. maxima*	*P. maxima* (AY877504.1) 492 bp (99 %)	KF284029	*yes*
BL4	9.2 / 1834	115	*P. margaritifera*	*P. margaritifera* (AY877506.1) 437 bp (100 %)	KF284030	***no***
BL5	9.1 / 1816	165	Not determined	Not determined		***no***
BL6	8.8 / 1754	146	*P. radiata*	*P. fucata* [e] (AB214478.1) 207 bp (100 %)	KF284031	*yes* *yes*
BL7	8.3 / 1658	417	*P. radiata*	*P. fucata* (AY877582.1) 538 bp (99 %)	KF284032	*yes*
BL8	7.8 / 1550	672	*P. margaritifera*	*P. margaritifera* [f]		*yes*
BL9	7.3 / 1456	178	*P. radiata*	*P. fucata* (AY877582.1) 511 bp (99 %)	KF284033	*yes*
BL10	6.8 / 1354	38	*P. margaritifera*	*P. margaritifera* [f]		*yes*
BL11	6.6 / 1324	547	*P. margaritifera*	*P. margaritifera* (AY877506.1) 437 bp (100 %)	KF284034	*yes*
BL12	6.0 / 1198	154	*P. margaritifera*	*P. margaritifera* (AY877506.1) 435 bp (100 %)	KF284035	*yes*
BL13	5.8 / 1154	615	*P. margaritifera*	*P. margaritifera* (AY877506.1) 437 bp (100 %)	KF284036	*yes*
BL14	4.3 / 868	142	*P. maxima*	*P. maxima* (AY877505.1) 494 bp (100 %)	KF284037	*yes*

BL15	4.3 / 856	84	*P. maxima*	*P. maxima* (AY877505.1) 494 bp (100 %)	KF284038	*yes*
BL16	4.1 / 818	184	*P. radiata*	*P. fucata* (AY877582.1) 489 bp (99 %)	KF284039	*yes*
BL17	3.3 / 652	170	*P. radiata*	*P. fucata* (AY877582.1) 538 bp (99 %)	KF284040	*yes*
BL18	2.5 / 504	88	*P. radiata*	*P. fucata* (AY877582.1) 489 bp (99 %)	KF284041	*yes*

[a] the sample used for DNA extraction consisted of material extracted from the interior of the pearl, including ground nacre and OM.

[b] ITS2: nuclear internal transcribed spacer 2 located between the 5.8S and 28S ribosomal RNA genes. RFLP: restriction fragment length polymorphism.

[c] *Pinctada* species assignment was based on the highest BLAST score (highest query coverage and maximal base pair identity). GenBank accession number shown in brackets.

[d] amplicon size (base pair) and maximal identity (%) of the sequence to the BLAST query.

[e] *P. fucata* is conspecific to *P. radiata* according to their ITS sequences [50].

[f] sample had low sequence quality, but the BLAST query in GenBank recovered the correct *Pinctada* species.
