# Supplementary material for: DNA Fingerprinting of Pearls to Determine Their Origins
Source: PLoS One. 2013 Oct 9;8(10):e75606. doi: 10.1371/journal.pone.0075606 (PMC3794040; doi:10.1371/journal.pone.0075606)
Supplement: Table S2 — PCR primers, amplicon lengths and references. (PDF) [file pone.0075606.s003.pdf]

Table S2. PCR primers, amplicon lengths and references.

Gene product	Organellar localization	Primer sequence (5'-3')	Primer name	Tann (°C)	Fragment length (bp)	Reference
16S rRNA [a]	Mitochondrial	CGCCTGGTTGATTAAAAACATTGCTGC	16S pinctada for	55	PMR: 511	[49], [50]
		CCGGTTTGAACTCAGATCACGTA	16S pinctada rev		PMX: 509	
					PR: 524	
cox1 [a]	Mitochondrial	TCGTATAGAGCTCCGTCGACCTG	LCX	45	PMR: 576	[45]
		TGGAACAAAACTGGATCGCC	HCY		PMX: 576	
					PR: 576	
ITS1 [a]	Nuclear	CACACCGCCCGTCGCTACTA	sp-1-5	52	PMR: 675	[44], [51]
		ATTTAGCTGCGGTCTTCATC	sp-1-3		PMX: 701	
					PR: 627	
ITS2 [a]	Nuclear	GCAGGACACATTGAACATCG	5.8S-F	52	PMR: 575	[51]
		CCAAGGACGTTCTTAGCAGAAG	28S-R		PMX: 571	
					PR: 590-591	
P. margaritifera ITS2 [a]	Nuclear	CTGTTCTGTCATGACGACGG	ITS2-Marg-F	52	PMR: 335	This study
P. maxima ITS2 [a]	Nuclear	GGGCCTATTTCCGTGTTGAG	ITS2-Max-F	52	PMX: 332	This study
P. radiata ITS2 [a]	Nuclear	CTGTCGATGGATGACTTACACG	ITS2-Rad-F	52	PR: 336-337	This study

[a] 16S rRNA: mitochondrial 16S ribosomal RNA gene; *cox1*: mitochondrial cytochrome oxidase subunit I gene; ITS1: nuclear internal transcribed spacer 1 located between the 18S and 5.8S ribosomal RNA gene; ITS2: nuclear internal transcribed spacer 2 located between the 5.8S and 28S ribosomal RNA genes.
